# Progress in RF-MEMS

**DOI:** 10.3390/mi16020233

**Published:** 2025-02-19

**Authors:** R. Niall Tait

**Affiliations:** Department of Electronics, Carleton University, Ottawa, ON K1S 5B6, Canada; niall.tait@carleton.ca

## 1. Introduction

As micro-electro-mechanical systems (MEMS) have evolved and matured over the last few decades, they have impacted a broad range of technologies and application areas. Very early in the development of MEMS, a high-Q frequency selective structure coupling a mechanical beam to the gate of a field effect transistor was proposed and demonstrated [1]. The field has since grown to establish a class of devices collectively referred to as radio-frequency MEMS (RF-MEMS), using micromechanical structures to manipulate electrical signals across a range of radio and microwave frequencies. These devices offer widely recognized advantages in a number of performance and use parameters; however, they also present practical challenges in reliability and packaging that have led to slower-than-expected commercial acceptance [2,3,4,5].

While there are a variety of uses for MEMS devices in RF and microwave applications, the majority of RF-MEMS fall into two main categories: resonators, both acoustic and mechanical [6,7,8], or switches and relays, both ohmic and capacitive [9,10,11,12,13]. These basic components are building blocks enabling compact low-power tunable filters and phase shifters essential in high-frequency systems [14,15,16].

It is likely that the period of below-expected market adoption experienced by RF-MEMS through most of the field’s history is now in the past [17]. Thin-film bulk acoustic resonator (FBAR) filters are well established as components in compact wireless systems, and demand is expected to continue growing [18]. Through steady improvements in materials and manufacturing [19], MEMS switches are moving towards increased integration [20] and gaining wider acceptance and improved reputation for reliability and power handling abilities [21].

## 2. Overview of Articles in Micromachines

RF-MEMS continues to be an active field of research with new materials, devices, and applications continually evolving in fields strongly overlapping with the scope of the journal *Micromachines*. Demand for multi-standard communication systems requires performance that can only be achieved through use of MEMS technology, and it is driving exciting research developments.

Due to both performance and processing characteristics, aluminum nitride (AlN) has been the preferred material for thin-film bulk acoustic resonator (FBAR) devices [22]. However, since Akiyama et.al. found that Scandium-doped AlN films could provide improved piezoelectric response [23], there has been great interest in developing fabrication processes that can leverage techniques already used for AlN-based devices.

The use of Sc-doped AlN as a piezoelectric material in radio-frequency MEMS applications is described in [24]. In addition to the improved piezoelectric coefficients, the ferroelectric-switching capability of Aluminum Scandium Nitride (Al_1−x_Sc_x_N) creates the possibility of integrating memory functionalities in RF components. Material properties still require some improvement, but there are a range of deposition parameters to be explored. In this work, the effect of RF substrate bias is investigated as a tool to help in engineering the Al_1−x_Sc_x_N lattice cell in order to modify leakage, breakdown, and coercive fields, as well as polarization charge.

The introduction of new materials can disrupt established fabrication process flows, and the successful adoption of a material often depends on related process challenges. In the case of Al_1−x_Sc_x_N thin films, resistance to etching at high scandium content has impeded the realization of devices with the highest electromechanical coupling. Relationships between scandium content and etching characteristics are explored in reference [25]. This work investigates the vertical and lateral etch rates of sputtered AlN and Al_1−x_Sc_x_N with Sc concentration x ranging from 0 to 0.42 in aqueous potassium hydroxide (KOH). Etch rates and the sidewall angles are reported at different temperatures and KOH concentrations. While the vertical etch rate is found to decrease with increasing Sc alloying, the lateral etch rate exhibits a V-shaped transition with a minimum etch rate at x = 0.125. Etch profile control is also explored by exploiting crystal-plane selective etch rates in KOH.

Application of piezoelectric materials such as AlN to radio-frequency filters depends on the key parameter of effective electromechanical coupling coefficient $k_{eff}^{2}$. In reference [26], the authors propose a method to tune $k_{eff}^{2}$ of a FBAR by etching the piezoelectric material to form a trench around the active area of the resonator. The trench parameters are investigated through both finite element modeling and fabricated devices. A circuit equivalent model is also presented for comparison to the physical model and measured results. This work suggests an approach to tuning $k_{eff}^{2}$ of resonators to satisfy filter requirements.

While FBAR devices have been widely used in filters for wireless systems, mechanical beam resonators have found applications typically at lower frequencies. Reference [27] examines, through simulation, a technique to improve the figure of merit of laterally vibrating RF-MEMS resonators. The technique uses a suspended addendum frame on the sides of the resonant plate acting as a mechanical vibration isolator from the supporting substrate. This enables the resonator to have low acoustic energy loss and a high quality factor.

Perhaps the most widely recognized RF-MEMS component is the micromechanical switch. While extensive research and development has gone into switch technology, it continues to evolve and improve. This includes improvements in materials, design, and packaging.

The design of an RF-MEMS device is a complex process, combining high-frequency electromagnetic design with electromechanical design, process design, and package or test-fixture design. Reference [28] describes a design methodology applied to the design of two single-pole single-throw switches intended for 5G wireless applications. The paper describes in detail the design flow including the choice of materials, simulation of electromechanical and RF behavior, device fabrication, and testing.

In reference [29], a high-isolation and high-capacitance-ratio RF-MEMS switch working at Ka-band is demonstrated. The proposed RF-MEMS switch configuration incorporates a MEMS metallic beam, coplanar waveguide (CPW) transmission line, dielectric layer, and metal–insulator–metal (MIM) fixed capacitors. The measured results show insertion loss better than 0.5 dB at 32 GHz, and isolation better than 35 dB at the resonant frequency with a switched capacitance ratio of 246.

While switch reliability has greatly improved since early implementations, this is still an active area of research. Reference [30] explores thermal effects in an RF-MEMS switch structure. In this paper, the Thermal–Mechanical-Stress-Creep (TMSC) effect during thermal processes from room temperature to 200 °C is modeled and measured for a Au-cantilever-based RF-MEMS switch. The authors describe isolation measurements that enable the extraction of capacitance and gap with attofarad and sub-nanometer resolution, respectively. The study looks at the thermal–mechanical stress in both anchor and cantilever, the grain growth of gold, and the thermal creep, which create competing effects during the heating and cooling of the cantilever.

One of the most important applications of RF-MEMS is in dynamically tunable filters. Reference [31] describes a bandpass filter based on a CPW capacitive switch. In this implementation, switch actuation couples a signal to a resonant ring providing the frequency selectivity. The authors discuss the benefits of compact size and good stopband rejection, insertion loss, and return loss provided by the integrated RF-MEMS filter.

In most applications, MEMS switches are integrated with additional passive components to enable more complex functions. Reference [32] describes an X-band phase detector implemented using a MEMS beam capacitor to couple power to a thermoelectric detector. This is not a conventional switch, but it does share many similar design requirements and illustrates potential for broad application and integration of RF-MEMS in monolithic microwave integrated circuits (MMICs). The phase detector described in this work considers the variable coupling due to actuation of the beam under RF power, and demonstrates superior power-handling and detection linearity while enabling a high level of integration.

Electronics research has begun to pursue printable, flexible, and conformal electronic technologies to enable wearable devices, the integration on curved surfaces, and a reduced environmental footprint. Most of these applications will benefit from wireless connections, and the integration of MEMS and RF-MEMS can support this capability. Reference [33] offers a review of advances in flexible RF-MEMS including switches, phase shifters, reconfigurable antennas, phased array antennas, and resonators. The authors discuss some of the challenges involved in accommodating flexible substrate deformation with the movable structures used in RF-MEMS. In addition, a fabrication process for reconfigurable three-dimensional RF devices based on mechanically guided assembly is introduced. The paper offers a useful reference source on advances in flexible RF-MEMS for researchers in this field.

## 3. Conclusions

Advances in RF-MEMS in recent years have been driven by improvements in materials, manufacturing techniques, integration with CMOS technologies, and the growing demand for more flexible, compact, and high-performance RF components. These advances have enabled the development of smaller, more reliable, and power-efficient systems that are essential to modern communication, defense, and aerospace technologies. This summary has reviewed only a sample of the interesting RF-MEMS research appearing in *Micromachines*; new papers in this field are continually appearing. It will be exciting to watch the continued evolution of MEMS with future applications involving even greater integration with emerging technologies, including advanced wireless, artificial intelligence and quantum communication systems.

## References

[1] Nathanson H.C., Newell W.E., Wickstrom R.A., Davis J.R. (1967). The resonant gate transistor. IEEE Trans. Electron Devices.

[2] Gaddi R., Gnudi A., Tazzoli A., Meneghesso G., Zanoni E. Reliability of RF-MEMS. Proceedings of the European Gallium Arsenide and Other Semiconductor Application Symposium, GAAS 2005.

[3] Tanner M. (2009). MEMS reliability: Where are we now?. Microelectron. Reliab..

[4] Saleem M.M., Nawaz H. (2019). A Systematic Review of Reliability Issues in RF-MEMS Switches. Micro Nanosyst..

[5] Jiang L., Ma N., Wang L., Huang X. (2023). High-reliability circular-contact RF MEMS switches in silicon hermetic package. J. Micromech. Microeng..

[6] Ng E., Yang Y., Hong V.A., Ahn C.H., Heinz D.B., Flader I., Chen Y., Everhart C.L.M., Kim B., Melamud R. The long path from MEMS resonators to timing products. Proceedings of the 2015 28th IEEE International Conference on Micro Electro Mechanical Systems (MEMS).

[7] Ruby R., Merchant P. Micromachined thin film bulk acoustic resonators. Proceedings of the IEEE 48th Annual Symposium on Frequency Control.

[8] Wu G., Xu J., Ng E.J., Chen W. (2020). MEMS Resonators for Frequency Reference and Timing Applications. J. Microelectromech. Syst..

[9] Rebeiz G.M. (2003). RF MEMS: Theory, Design, and Technology.

[10] Rebeiz G.M., Muldavin J.B. (2001). RF MEMS switches and switch circuits. IEEE Microw. Mag..

[11] Kurmendra, Kumar R. (2021). A review on RF micro-electro-mechanical-systems (MEMS) switch for radio frequency applications. Microsyst. Technol..

[12] Shao B., Lu C., Xiang Y., Li F., Song M. (2024). Comprehensive Review of RF MEMS Switches in Satellite Communications. Sensors.

[13] Percy J.J., Kanthamani S. (2023). Revolutionizing wireless communication: A review perspective on design and optimization of RF MEMS switches. Microelectron. J..

[14] Iannacci J., Tagliapietra G., Bucciarelli A. (2022). Exploitation of response surface method for the optimization of RF-MEMS reconfigurable devices in view of future beyond-5G, 6G and super-IoT applications. Sci. Rep..

[15] Deng Z., Wang Y., Lai C. (2023). Design and Analysis of Pattern Reconfigurable Antenna Based on RF MEMS Switches. Electronics.

[16] Iannacci J., Tagliapietra G. (2022). Getting ready for beyond-5G, super-IoT and 6G at hardware passive components level: A multi-state RF-MEMS monolithic step attenuator analyzed up to 60 GHz. Microsyst. Technol..

[17] Iannacci J. (2017). Expectations versus actual market of RF-MEMS: Analysis and explanation of a repeatedly fluctuating scenario. RF-MEMS Technology for High-Performance Passives.

[18] Hagelauer A., Ruby R., Inoue S., Plessky V., Hashimoto K.-Y., Nakagawa R., Verdu J., de Paco P., Mortazawi A., Piazza G. (2023). From Microwave Acoustic Filters to Millimeter-Wave Operation and New Applications. IEEE J. Microw..

[19] Kurmendra, Kumar R. (2021). Materials Selection Approaches and Fabrication Methods in RF MEMS Switches. J. Electron. Mater..

[20] Göritz A., Wipf S.T., Drost M., Lisker M., Wipf C., Wietstruck M., Kaynak M. (2022). Monolithic Integration of a Wafer-Level Thin-Film Encapsulated mm-Wave RF-MEMS Switch in BEOL of a 130-nm SiGe BiCMOS Technology. IEEE Trans. Compon. Packag. Manuf. Technol..

[21] Zorpette G. (2020). RF MEMS deliver the “ideal switch”: After two decades of development, MEMS-based RF switches are finally finding real-world uses. IEEE Spectr..

[22] Li J., Chen Z., Liu W., Yang J., Zhu Y., Yang F. (2022). A novel piezoelectric RF-MEMS resonator with enhanced quality factor. J. Micromech. Microeng..

[23] Akiyama M., Kamohara T., Kano K., Teshigahara A., Takeuchi Y., Kawahara N. (2009). Enhancement of piezoelectric response in scandium aluminum nitride alloy thin films prepared by dual reactive cosputtering. Adv. Mater..

[24] Pirro M., Zhao X., Herrera B., Simeoni P., Rinaldi M. (2022). Effect of Substrate-RF on Sub-200 nm Al0.7Sc0.3N Thin Films. Micromachines.

[25] Tang Z., Esteves G., Zheng J., Olsson R. (2022). Vertical and Lateral Etch Survey of Ferroelectric AlN/Al1−xScxN in Aqueous KOH Solutions. Micromachines.

[26] Gao C., Zou Y., Zhou J., Liu Y., Liu W., Cai Y., Sun C. (2022). Influence of Etching Trench on K_eff_^2^ of Film Bulk Acoustic Resonator. Micromachines.

[27] Workie T., Wu Z., Tang P., Bao J., Hashimoto K. (2022). Figure of Merit Enhancement of Laterally Vibrating RF-MEMS Resonators via Energy-Preserving Addendum Frame. Micromachines.

[28] Tkachenko A., Lysenko I., Kovalev A. (2023). Investigation and Research of High-Performance RF MEMS Switches for Use in the 5G RF Front-End Modules. Micromachines.

[29] Deng Z., Wang Y., Deng K., Lai C., Zhou J. (2022). Novel High Isolation and High Capacitance Ratio RF MEMS Switch: Design, Analysis and Performance Verification. Micromachines.

[30] Zhang Y., Sun J., Liu H., Liu Z. (2022). Modeling and Measurement of Thermal–Mechanical-Stress-Creep Effect for RF MEMS Switch Up to 200 °C. Micromachines.

[31] Zhang Y., Cui M., Wu D. (2023). Design and Fabrication of a MEMS Bandpass Filter with Different Center Frequency of 8.5–12 GHz. Micromachines.

[32] Han J., Ding D. (2022). Design and Analysis of a Hybrid-Type RF MEMS Phase Detector in X-Band. Micromachines.

[33] Shi Y., Shen Z. (2022). Recent Advances in Flexible RF MEMS. Micromachines.

